# Melatonin and/or erythropoietin combined with hypothermia in a piglet model of perinatal asphyxia

**DOI:** 10.1093/braincomms/fcaa211

**Published:** 2020-12-01

**Authors:** Raymand Pang, Adnan Avdic-Belltheus, Christopher Meehan, Kathryn Martinello, Tatenda Mutshiya, Qin Yang, Magdalena Sokolska, Francisco Torrealdea, Mariya Hristova, Alan Bainbridge, Xavier Golay, Sandra E Juul, Nicola J Robertson

**Affiliations:** 1 Department of Neonatology, Institute for Women's Health, University College London, London, UK; 2 Department of Medical Physics and Biomedical Engineering, University College London Hospitals, London, UK; 3 Department of Brain Repair and Rehabilitation, Institute of Neurology, Queen’s Square, University College London, London, UK; 4 Department of Pediatrics, University of Washington, Seattle, Washington, DC, USA

**Keywords:** neonatal encephalopathy, neuroprotection, melatonin, erythropoietin, therapeutic hypothermia

## Abstract

As therapeutic hypothermia is only partially protective for neonatal encephalopathy, safe and effective adjunct therapies are urgently needed. Melatonin and erythropoietin show promise as safe and effective neuroprotective therapies. We hypothesized that melatonin and erythropoietin individually augment 12-h hypothermia (*double* therapies) and hypothermia + melatonin + erythropoietin (*triple therapy*) leads to optimal brain protection. Following carotid artery occlusion and hypoxia, 49 male piglets (<48 h old) were randomized to: (i) hypothermia + vehicle (*n* = 12), (ii) hypothermia + melatonin (20 mg/kg over 2 h) (*n* = 12), (iii) hypothermia + erythropoietin (3000 U/kg bolus) (*n* = 13) or (iv) *tripletherapy* (*n* = 12). Melatonin, erythropoietin or vehicle were given at 1, 24 and 48 h after hypoxia–ischaemia. Hypoxia–ischaemia severity was similar across groups. Therapeutic levels were achieved 3 hours after hypoxia–ischaemia for melatonin (15–30 mg/l) and within 30 min of erythropoietin administration (maximum concentration 10 000 mU/ml). Compared to hypothermia + vehicle, we observed faster amplitude-integrated EEG recovery from 25 to 30 h with hypothermia + melatonin (*P* = 0.02) and hypothermia + erythropoietin (*P* = 0.033) and from 55 to 60 h with *tripletherapy* (*P* = 0.042). Magnetic resonance spectroscopy lactate/*N*-acetyl aspartate peak ratio was lower at 66 h in hypothermia + melatonin (*P* = 0.012) and *tripletherapy* (*P* = 0.032). With hypothermia + melatonin, terminal deoxynucleotidyl transferase-mediated deoxyuridine triphosphate nick-end labelled-positive cells were reduced in sensorimotor cortex (*P* = 0.017) and oligodendrocyte transcription factor 2 labelled-positive counts increased in hippocampus (*P* = 0.014) and periventricular white matter (*P* = 0.039). There was no reduction in terminal deoxynucleotidyl transferase-mediated deoxyuridine triphosphate nick-end labelled-positive cells with hypothermia + erythropoietin, but increased oligodendrocyte transcription factor 2 labelled-positive cells in 5 of 8 brain regions (*P* < 0.05). Overall, melatonin and erythropoietin were safe and effective adjunct therapies to hypothermia. Hypothermia + melatonin *double therapy* led to faster amplitude-integrated EEG recovery, amelioration of lactate/N-acetyl aspartate rise and reduction in terminal deoxynucleotidyl transferase-mediated deoxyuridine triphosphate nick-end labelled-positive cells in the sensorimotor cortex. Hypothermia + erythropoietin *doubletherapy* was in association with EEG recovery and was most effective in promoting oligodendrocyte survival. *Tripletherapy* provided no added benefit over the *double* therapies in this 72-h study. Melatonin and erythropoietin influenced cell death and oligodendrocyte survival differently, reflecting distinct neuroprotective mechanisms which may become more visible with longer-term studies. Staggering the administration of therapies with early melatonin and later erythropoietin (after hypothermia) may provide better protection; each therapy has complementary actions which may be time critical during the neurotoxic cascade after hypoxia–ischaemia.

## Teaser

As therapeutic hypothermia is partially protective in neonatal encephalopathy, adjunct therapies are required. In a piglet model of perinatal asphyxia, Pang *et al.* report that melatonin and erythropoietin are safe and individually augment cooling through different mechanisms. Staggered rather than concomitant dosing may be optimal for brain protection.

## Graphical Abstract

**Graphical Abstract:**
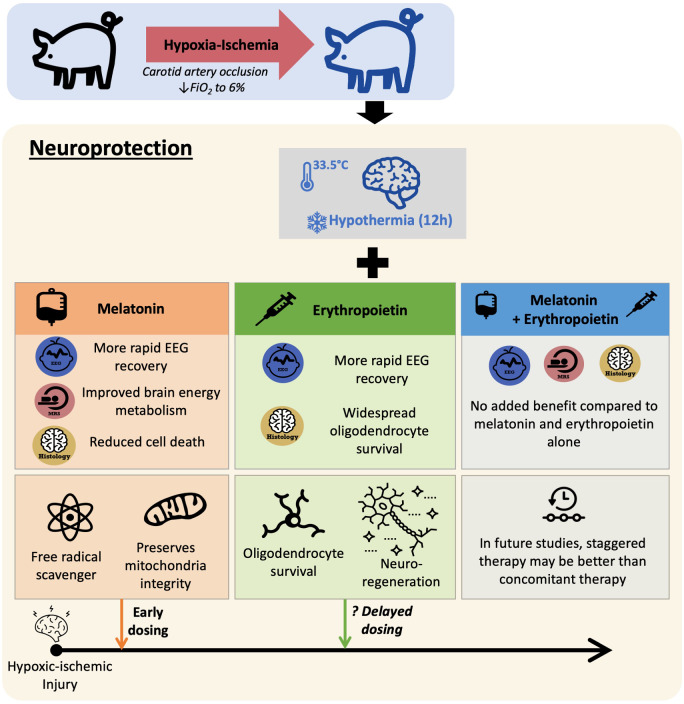

## Introduction

Intrapartum-related hypoxia–ischaemia (HI) leading to neonatal encephalopathy (NE) is a major burden for the individual, family and society. The incidence of NE varies across the world, affecting 1–3.5/1000 live births in high-resource settings (Gale *et al.*, 2018; Royal College of Obstetricians & Gynaecologists, 2018) and ∼26 per 1000 in low-resource settings (Lee *et al.*, 2013). Therapeutic hypothermia (HT) is currently the only treatment for NE and is routinely used in high-resource settings (Tagin *et al.*, 2012). While HT is clearly beneficial with a number needed to treat to prevent death or disability of 7 (Tagin *et al.*, 2012), infants still experience unacceptably high complications. Recent UK data show that, with the current practice of HT, the mortality of NE has reduced from 25% to 9% in clinical trials, while disability dropped from 20% to ∼16% with a reduction in the rate of cerebral palsy (Jary *et al.*, 2015). It is clear that not all children benefit from treatment and some level of intellectual impairment may remain even in the absence of cerebral palsy (Lee-Kelland *et al.*, 2020). Further attempts to refine HT suggest current cooling protocols are optimal (Davies *et al.*, 2019) and adjunct therapies are needed to improve neonatal outcomes (Robertson *et al.*, 2012).

Over the past decade, melatonin [*N*-acetyl-5-methoxytryptamine (MEL)] and erythropoietin (Epo) have shown promise as safe and effective neuroprotective agents with the potential to augment hypothermic brain protection in pre-clinical (Fan *et al.*, 2013; Robertson *et al.*, 2013; Traudt *et al.*, 2013; Carloni *et al.*, 2018; Robertson *et al.*, 2019; Robertson *et al.*, 2020) and clinical studies (Wu *et al.*, 2012; Wu *et al.*, 2016). MEL has antioxidant, anti-apoptotic, anti-excitatory and anti-inflammatory properties which act through receptor and non-receptor-dependent pathways (Ramos *et al.*, 2017). In our piglet model, augmentation of 24 h HT with intravenous 30 mg/kg MEL dissolved in ethanol, started 10 min and repeated at 24 h after HI was safe and improved amplitude-integrated electroencephalogram (aEEG) brain recovery, brain energy metabolism on magnetic resonance spectroscopy (MRS) and reduced cell death (Robertson *et al.*, 2013). As MEL is sparingly water-soluble, excipients or solubility enhancers are needed. Ethanol is frequently used as a solubility enhancer in clinical (Fulia *et al.*, 2001) and pre-clinical studies (Yawno *et al.*, 2012; Robertson *et al.*, 2013; Drury *et al.*, 2014; Aridas *et al.*, 2018; Robertson *et al.*, 2020) but recently the ethanol excipient has been shown to influence cell death (Drury *et al.*, 2014; Robertson *et al.*, 2019) and may not be safe for neonatal use. In the present study, we further modified our treatment regimen using an ethanol-free, highly concentrated (5 mg/ml) proprietary MEL formulation developed using excipients considered safe for use in newborns (Int. pat. appl. PCT/EP2018/056423; https://patentscope.wipo.int/search/en/detail.jsf?docId=WO2018167162). Our pharmacokinetic (PK) and efficacy studies with MEL suggest that, for optimal protection, therapeutic levels of 15–30 mg/l MEL are needed soon after HI (Robertson *et al.*, 2013, 2019, 2020). Our PK modelling defined a dose of 20 mg/kg given as an infusion over 2 h, started 1 h after HI as the optimal dose.

Epo has anti-inflammatory, anti-excitotoxic, antioxidant and anti-apoptotic (Rangarajan and Juul, 2014; Juul and Pet, 2015) effects on neurons and oligodendrocytes. Long-term regenerative effects include neurogenesis (Gonzalez *et al.*, 2013), oligodendrogenesis (Jantzie *et al.*, 2013) and angiogenesis (Wang *et al.*, 2008). Clinical trials (University of Sydney National Health and Medical Research Council Australia, 2016; Juul *et al.*, 2018) are underway to evaluate the safety and efficacy of Epo as an adjunct to HT in NE. The phase I study demonstrated intravenous Epo at 1000 U/kg was well tolerated and achieved putative therapeutic levels previously shown in animal studies (Wu *et al.*, 2012). Improved short term motor outcomes and reduced brain injury on magnetic resonance imaging (MRI) were seen with Epo in combination with HT in a small phase II trial (Wu *et al.*, 2016).

In this 72 h study, we hypothesized: (i) MEL (optimized dose and infusion rate with target therapeutic levels at 3 h after HI) would augment HT (33.5°C for 12 h, started 1 h after HI) *(double therapy)*; (ii) HT + Epo, investigated in our lab for the first time with doses achieving similar target therapeutic levels as in clinical trials, would augment HT *(double therapy)*; and (iii) Combined HT, MEL and Epo (*triple therapy*) would provide best brain protection. Primary outcome measures were: (i) aEEG background activity recovery over 48 h, a strong predictor of outcome in babies with NE (Thoresen *et al.*, 2010); (ii) proton (^1^H) MRS lactate/*N*-acetyl aspartate (Lac/NAA), a robust and accurate neurodevelopmental outcome biomarker at 2 years (Mitra *et al.*, 2019) and (iii) immunohistochemical assessment of cell death using terminal deoxynucleotidyl transferase-mediated deoxyuridine triphosphate nick-end labelling (TUNEL-positive cells) and oligodendrocyte survival (OLIG2-positive cells) in eight brain regions at 72 h after HI.

## Materials and methods

The study was approved by the ethics committee of University College London, conducted according to UK Home Office Regulations [Animals (Scientific procedures) Act, 1986] and complies with the ARRIVE guidelines (Percie Du Sert *et al.*, 2020).

### Sample size

In a previous MEL study (Robertson *et al.*, 2013) in our piglet model, a treatment difference for Lac/NAA of 0.77 log units with a standard deviation estimate of 0.37 was observed. In the study of Argon^31^, we observed a difference in the Lac/NAA at 48 h of 0.5 log units and an standard deviation estimate of 0.3–0.35. Based on standard deviation of 0.4 and an expected difference between groups of single therapy plus cooling versus cooling alone of 0.5, we need 12 piglets in each group to detect significance with 95% confidence at 80% power.

### Protocol

**Figure 1:**
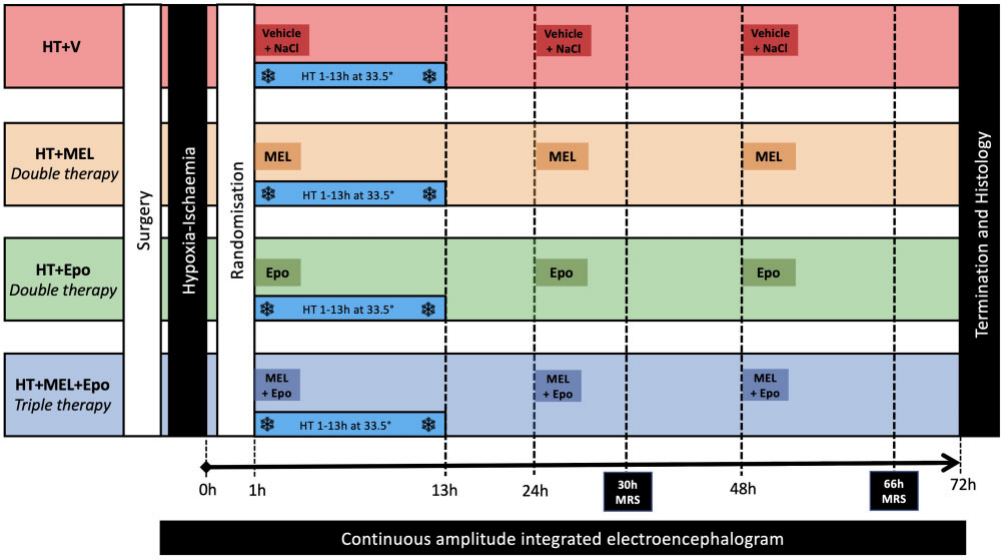
**Study protocol.** Baseline vital signs were taken prior to induction of anaesthesia and surgical preparation. Following surgery, all piglets underwent cerebral HI by inflation of carotid occluders and reduction of inspired oxygen to 6%. Piglets were resuscitated after HI and observed for 1 h prior to randomization to (i) HT + vehicle (HT+V), (ii) HT+MEL (MEL) *double therapy*, (iii) HT + Epo *double therapy* or (iv) HT+MEL+Epo *triple therapy*. All piglets were cooled to 33.5°C for 12 h from 1 h after HI. MEL 20 mg/kg was infused intravenously over 2 h and Epo 3000 units/kg was given as an intravenous bolus. These were given at 1 h, 24 h and 48 h after HI. The HT+V group received vehicle infusion at an equivalent volume and rate as MEL and a bolus of 0.9% sodium chloride at the same volume as Epo. MRS was acquired out-of-hours at 30 h (18:00) and 66 h (06:00) using the clinical 3 T *Philips Achieva* magnetic resonance scanner. Cerebral electrical activity was continuously monitored with aEEG. Studies were terminated at 72 h.

The experimental protocol is summarized in Fig. 1. The model has been modified since the first description (Lorek *et al.*, 1994); our recent modifications include longer experimental duration of 72 h from 48 h, a cooling period of 12 h from 1 to 13 h after HI, continuous aEEG/EEG (Nicolet, Care Fusion, Wisconsin, USA) monitoring throughout the study, cerebral HI titration according to blood gas and mean arterial blood pressure (MABP) response rather than phosphorus-31 MRS and the transfer to a clinical 3 T magnetic resonance system (Philips *Achieva*) twice during the study. Our previous piglet studies have used 24 h HT protocols (Robertson *et al.*, 2013, 2019) which show protection compared to normothermia (Iwata *et al.*, 2005; Alonso-Alconada *et al.*, 2015). More recently, we have used 12 h HT protocols (Lingam *et al.*, 2019; Robertson *et al.*, 2020) to allow modelling of the serial events of cooling, rewarming and a period of normothermia after HI, as in the clinical setting. This protocol allows for MRS data acquisition over two time points at normothermia (as with babies with NE) following the controlled rewarming phase.

### Surgery

In brief, newborn male Large White Piglets, aged <48 h, 1.6–2.2 kg in weight, were sedated with intramuscular midazolam (0.2 mg/kg) and anaesthetized with isoflurane mixed with air (3% v/v during surgery, 1.5–2.5% during experimentation), remaining insentient throughout experimentation. Animals were mechanically ventilated via tracheostomy (SLE 2000 infant ventilator, Surrey, UK) and settings guided by arterial blood gas analysis (PaO_2_ 8–13 kPa, pCO_2_ 4.5–6.5 kPa). The common carotid arteries were encircled by inflatable carotid occluders (OC2A, In Vivo Metric). An umbilical arterial line was inserted for MABP monitoring and umbilical venous line for infusions. Infusions included maintenance 10% dextrose at 60 ml/kg/day (reduced to 40 ml/kg/day post-HI), fentanyl 3 mcg/kg/h, and antibiotics (benzylpenicillin 50 mg/kg/dose BD, gentamicin 5 mg/kg/dose OD). The arterial line was infused with heparinized saline (0.5 IU/ml in 0.9% sodium chloride) at 0.3 ml/h. Animals were nursed prone in a purpose-built MRI-compatible transport incubator. Intensive care was provided throughout the 72 h experiment and complications (e.g. hypotension, seizures, hyperkalaemia) were treated as per local neonatal guidelines. To maintain the MABP >40 mmHg, dopamine (5–20 mcg/kg/min), dobutamine (5–20 mcg/kg/min), noradrenaline (0.1–1.5 mcg/kg/min) and adrenaline (0.1–1.5 mcg/kg/min) were used as required.

### Transient cerebral HI

Cerebral HI was commenced by simultaneously inflating the carotid occluders remotely and reducing the fraction of inspired oxygen to 6% over the first 3 min as previously described (Robertson *et al.*, 2020). To achieve a uniform HI insult, two experienced team members determined the exact length of HI in real-time using a strict protocol. We targeted an overall duration of between 20–25 min, achieving aEEG <5 mV for 15–20 min. Fraction of inspired oxygen was titrated up by 1% every minute in response to MABP <25 mmHg and titrated down by 1% every minute if MABP was >30 mmHg. HI insult was terminated once we achieved blood lactate between 10 mmol/l and 12 mmol/l or we observed severe refractory hypotension (MABP < 25mmHg) not responding to increased fraction of inspired oxygen, suggesting imminent cardiac arrest. At the end of the insult, the animal was resuscitated, occluders deflated and fraction of inspired oxygen increased to air.

### Randomization

Eligibility criteria to enter the study were: (i) normal baseline aEEG following surgery (ii) no recovery in baseline aEEG within the first hour following HI and (iii) no pyrexia (Temperature >39°C). Refractory hypotension after HI resistant to inotropic support was an *a priori* exclusion criteria. Randomization was by computer-generated randomization into 4 groups; this was performed after the end of HI. Subgroups of six piglets were block randomized to ensure equal spread across the 2-year study. Only male piglets were used.

### Neuroprotective intervention

Rectal temperature was maintained at 38°C by a servo-controlled water mattress (Tecotherm). During HT, piglets were cooled to 33.5°C for 12 h followed by controlled rewarming at 0.5°C/h. HT target temperature was achieved within a median of 45 min (interquartile range 45–60 min). MEL (Chiesi Pharmaceuticals, Italy Int. pat. appl. PCT/EP2018/056423) 20 mg/kg was infused over 2 h starting at 1 h, 24 h and 48 h after HI. Epo (Eprex, Janssen-Cilag, UK) 3000 U/kg was administered as an intravenous bolus at 1 h, 24 h and 48 h. HT+V animals received a vehicle infusion (excipient used for MEL solubility, Chiesi Pharmaceuticals, Parma, Italy) at the equivalent rate and volume as MEL for weight plus a bolus of 0.9% sodium chloride at the same volume as Epo.

### Amplitude-integrated electroencephalogram

Following surgery, continuous multichannel six-lead amplitude EEG monitoring (Nicolet, Care Fusion, Wisconsin, USA) was initiated. Hourly aEEG scores were classified according to (Hellstrom-Westas *et al.*, 1995) by a clinician (R.P.) and an experienced lab technician (C.M.) blinded to treatment group and averaged over 6 h intervals. Electrographic seizures were recorded and treated with phenobarbitone 20 mg/kg loading over 20 min followed by a further 10 mg/kg dose if seizures persisted.

### Magnetic resonance imaging

Magnetic resonance imaging (MRI) was acquired ‘out-of-hours’ at 30 h and 66 h using the Philips Achieva 3 T scanner (Phillips, Netherlands). Imaging protocols were similar to those used in babies with NE (Mitra *et al.*, 2019). MRS using chemical shift imaging was performed with a repetition time (TR) of 2 s and echo time (TE) of 288 ms. Voxels were 8 × 8 × 10 mm^3^ over an 8 × 8 matrix. The spectral width was 2 kHz with 2048 points. Data from the voxels over the left basal ganglia and thalamus (BGT) and the left subcortical white matter (WM) at the level of centrum semiovale were selected and processed using the Open-Source Tarquin (http://tarquin.sourceforge.net/) by a physicist blinded to treatment group. The Lac/NAA peak area ratio represents lactate+threonine/total *N*-acetyl aspartate + *N*-acetylaspartylglutamate, as the inclusion of threonine and total NAA in the spectral fitting has been shown to optimize accurate prediction of neurodevelopmental outcome in babies with NE (Mitra *et al.*, 2019).

### Immunohistochemistry

Piglets were euthanized at 72 h with intravenous pentobarbital and the brain dissected and stored in 2% paraformaldehyde. Two coronal slices (5 mm thick) from the right hemisphere at the level of the optic chiasm and hippocampus (HIP) were embedded in paraffin and cut into 8 µm sections. Prior to immunohistochemistry staining, sections were dehydrated in xylene and rehydrated in graded ethanol solution (100–70%).

Transferase-mediated biotinated deoxyuridine triphosphate nick-end labelled (TUNEL) was used to assess cell death over eight regions of the brain from both sections. Slices were treated with 3% hydrogen peroxide followed by pre-digestion with protease K (Promega, Southampton, UK) and incubated in TUNEL solution for 2 h (Roche, Burgess Hill, UK). Slices were then incubated in avidin-biotinylated horseradish peroxidase complex (ABC, Vector Laboratories) followed by diaminobenzidine/H_2_O_2_ (Sigma) with CoCl_2_ and NiCl_2_. A haematoxylin–eosin counter stain was applied, and slices were mounted on coverslips with DPX. TUNEL-positive cells were counted in three fields at 40× magnification for each of the eight regions of the brain at two levels. TUNEL counts were averaged for each subject and for each brain region across all fields and then scaled up to cells per mm^2^.

For immunohistochemistry, slides were pre-treated with Ventana cell conditioning solution (950–124) for OLIG2 and protease 1 (0.338 mg/ml alkaline protease enzyme activity) for glial fibrillary acidic protein (GFAP). Sections were incubated with primary rabbit antibody against oligodendrocyte transcription factor 2 (OLIG2, 1:500; Millipore) for 4 h and GFAP (1:1000, DAKO Z0334) for 32 min followed by incubation in swine anti-rabbit secondary antibodies (DAKO E03433) for 1 h in OLIG2 and 32 min in GFAP. Sections were subsequently stained with avidin-biotinylated horseradish peroxidase, diaminobenzidine/H_2_O_2_ and CoCl_2_ and NiCl_2_ for TUNEL visualization as mentioned earlier. Slides were dehydrated in graded alcohol and mounted on 4′,6-diamidino-2-phenylindole (DAPI) aqueous mounting media (Vector Labs). Similar to TUNEL, OLIG2 positive cells were counted in three fields at 40× magnification for each region, then averaged out per region and scaled up to cells per mm^2^. GFAP luminosity was deduced by deducting mean brightness values of tissues from the mean brightness of a blank region of the slide at 20× magnification, then averaged out over the three fields per region.

Immunohistochemistry examination was carried out by an investigator (CM) blinded to treatment group. For each animal, TUNEL, GFAP and OLIG2 counts over eight regions of the brain were examined over three fields, (Supplementary Fig. 1) across two sections (bregma 00 and −2.0) and averaged per brain region. This provides the best compromise between the amount and accuracy of the data and time taken to produce this.

### Pharmacokinetic analysis

Serum EPO levels were detected using the U-Plex Human EPO Assay (Meso Scale Discovery, KV151VXK). Blood serum samples were prepared according to the manufacturer’s instructions and plates were read using the sector imager 2400. Levels of MEL were measured using a validated ultraperformance liquid chromatography-tandem mass spectroscopy method in the range from 50 to 2000 ng/ml. Due to the rare matrix of the study (piglet serum) and since the expected MEL levels in the range 1–60 µg/ml, piglet serum samples were 100-fold diluted using commercially available mini-pig plasma and consequently the validation was performed in mini-pig plasma; stability and dilution effect were tested in piglet serum using a pool of baseline and T = 0 samples from the study in order to reproduce real-life conditions. Diluted samples were extracted by protein precipitation using 200 µl of acetonitrile containing deuterated MEL as internal standard and quantified on a calibration curve prepared using a blank matrix. The accuracy of the quantitation was verified using quality controls prepared in the same matrix and backcalculated on the curve. Bioanalysis was performed using an Acquity UPLC (Waters) coupled with an API 3200 Triple Quadrupole (ABSciex). Chromatography separation was achieved using a Kinetex XB C18 50 mm × 2.1 mm column (Phenomenex) under linear-gradient conditions from 2% to 98% solvent B in 1 min (phase A: Water + 0.1% formic acid; phase B: Acetonitrile + 0.1% Formic acid; temperature: 35°C; flow: 0.3 ml/min; injection volume: 5 μl). The mass spectrometer was operated in positive Electrospray Ionization mode and quantification was achieved by multiple reaction monitoring mode (transitions 233.5→174.2/159.0/143.0 and 237.1→178.2/163.1 for MEL and deuterated MEL respectively; source conditions: Gas 1: 25; Gas 2: 35; collision-activated dissociation gas 5; curtain gas 30; Source T 450°C; IS 5500). Plots of the calibration curve, the slope, the intercept values and correlation coefficients (R) concentrations values and accuracy were directly determined by Analyst 1.6.3 Software. Mean value, standard deviation and coefficient of variation values were calculated with Microsoft Office Excel 2010 software. Area under the curve (AUC) 0–72 h was calculated for each individual profile (method Linear Trapezoidal; Excel Add –in).

### Data and statistical analysis

Analysis was performed using Prism v8 (GraphPad Software LCC, USA) and SAS JMPv14. Graphical methods were used to assess the distribution of the results. Due to the skewed distribution of Lac/NAA peak ratio data, TUNEL-positive cell counts and OLIG2 cell counts, data were log_10_ transformed as standard practice. A factor of 0.5 was added prior to taking logged values to enable the inclusion of zero values, where present in the analyses. Comparison between treatment groups for physiological data, haematology results, aEEG, MRS and immunohistochemistry were made using analysis of variance (ANOVA) modelled with fixed factor effects of treatment, time interval and treatment*time interval interaction, plus a random effect subject to take account of repeated measures. *Post hoc* comparisons between treatment groups were assessed using 95% confidence intervals for the difference in the least square means and *P* values (two-tailed significance) for overall treatment differences and treatment differences within regions. Results were graphically presented using least square means and standard error of the means (SEM). TUNEL and Lac/NAA peak ratios were presented on log_10_ scale due to the influence of higher values in some of the data. OLIG2 data was back transformed to give geometric means and ratio of geometric means to allow comparison using the original cell/mm^2^ scale. Due to the exploratory nature of this study, no adjustment was made for multiplicity. As inotrope dose requirements were extremely skewed, differences between groups were presented as medians and interquartile ranges and assessed using Kruskal–Wallis Test.

### Data availability

The data that support the findings of this study are available from the corresponding author, upon reasonable request.

## Results

Piglets were 40.5 h old (range 24–53 h) at the start of the study. Two piglets were excluded before HI due to pyrexia (*n* = 1) and failure to obtain arterial access (*n* = 1). Five animals were excluded following HI due to rupture of vascular occluders (*n* = 3), aEEG recovery within 1 h of HI indicating a mild insult (*n* = 1) and HI physiological parameters outside the target range (*n* = 1). Four piglets died at <24 h due to refractory hypotension resistant to treatment (HT+MEL *n* = 1, HT+Epo *n* = 3) so their primary outcome data were not available. One piglet in HT+MEL group was excluded a priori for severe, refractory hypotension with lactic acidosis and subsequent secondary cerebral deterioration on aEEG. Forty-nine animals were available for primary outcome analysis: (i) HT+V (*n* = 12), (ii) HT+MEL *double therapy* (*n* = 12), (iii) HT+Epo *double therapy* (*n* = 13) and (iv) HT+MEL+Epo *triple therapy* (*n* = 12).

### Insult severity and physiological parameters

There were no intergroup differences in weight and physiological parameters (*P* > 0.05) (Supplementary Table 1). We observed no intergroup differences in HI insult severity (duration of insult; hypotension and isoelectric background activity on aEEG) (Table 1). Post-HI blood gas (Table 1) results were similar across the groups except the base excess was lower in the HT+V group compared to HT+MEL [Least square (LS) means difference 3.42, 95% CI (0.3–6.5), *P* = 0.03] and HT+MEL+Epo [difference 3.9, 95% CI (0.83–7.0), *P* = 0.013]. Base excess was higher in HT+MEL at 24 h compared to HT+V and HT+Epo [difference 4.5, 95% CI (1.4–7.6), *P* = 0.005]. A difference in pH at 24 h between groups (*P* = 0.033) was observed however pairwise analysis did not reveal a significant difference. No differences in inotropic requirements were observed between the treatment groups (Supplementary Table 1).

**Table 1:** Physiological parameters of hypoxic-ischaemic (HI) insult

		HT + V		HT + MEL		HT + Epo		HT + MEL + Epo		*P* value
		LS mean	95% CI	LS mean	95% CI	LS mean	95% CI	LS mean	95% CI	
Duration of HI (min)		23.3	(21.8, 24.7)	22.5	(21.0, 24.0)	22.6	(21.2, 24.0)	21.8	(20.3, 23.2)	0.556
Duration of EEG score 0 (min)		20.7	(19.5, 21.9)	20.0	(18.9, 21.3)	20.1	(18.9, 21.3)	19.6	(18.4, 20.8)	0.660
Duration of MABP <30 mmHg (min)		10.8	(8.6, 12.9)	9.2	(7.0, 11.4)	12.3	(10.2, 14.4)	11.0	(8.8, 13.2)	0.248
Duration of MABP <25 mmHg (min)		3.3	(1.6, 4.9)	3.2	(1.5, 4.9)	3.6	(2.0, 5.2)	4.2	(2.5, 5.9)	0.837
End of HI blood gas results										
pH		7.23	(7.18, 7.27)	7.29	(7.24, 7.33)	7.27	(7.23, 7.32)	7.30	(7.26, 7.34)	0.172
pCO_2_ (kPa)		5.8	(5.2, 6.4)	5.7	(5.0, 6.3)	5.4	(4.8, 6.0)	5.6	(4.9, 6.2)	0.834
pO_2_ (kPa)		4.0	(2.1, 5.9)	4.0	(2.1, 5.9)	4.1	(2.2, 5.9)	4.6	(2.7, 6.5)	0.586
Base excess (mEq/l)		−9.8	(−12.0, −7.6)	−6.4	(−8.6, −4.2)	−8.2	(−10.3, −6.1)	−5.9	(−8.1, −3.7)	**0.01**
Lactate (mmol/l)		12.7	(11.9, 13.5)	11.5	(10.7, 12.3)	11.0	(10.3, 11.8)	11.7	(10.9, 12.5)	0.054
Glucose (mmol/l)		9.9	(8.0, 11.8)	9.9	(7.8, 11.9)	9.3	(7.4, 11.3)	9.3	(7.2, 11.5)	0.938

An ANOVA model was fitted to each group and least square means (LS means), 95% confident intervals and one-way ANOVA *P* values are shown. Statistical significance (*P* < 0.05) highlighted in bold.

### Electroencephalography

**Figure 2:**
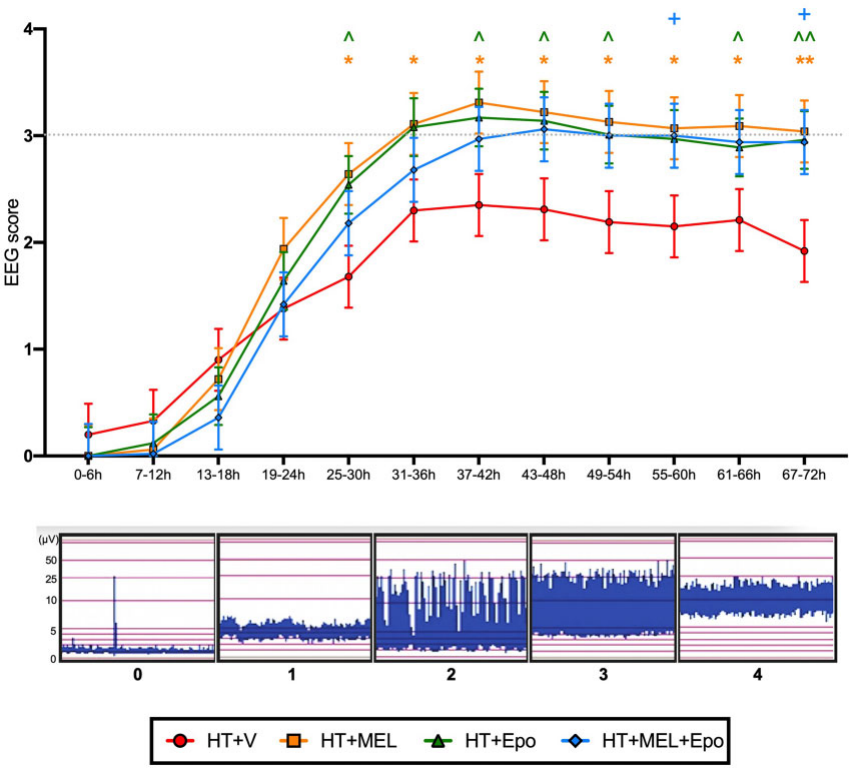
**aEEG.** Hourly aEEG activity was classified according to Hellstrom-Westas *et al.* (1995) and scores averaged over 6 h intervals. Data presented are the grouped least square means aEEG scores ± standard error of the means (SEM). The least square means was derived from an ANOVA model fitted with fixed factor effects of treatment, time interval and treatment*time interval interaction, plus a random effect subject to take account of repeated measures. Comparison between treatment groups were assessed using 95% confidence intervals for difference in the least square means and *P* values. Statistical significance when compared to HT+V as shown: *(HT+MEL), ^(HT+Epo) and ^+^(HT+MEL+Epo) where *P* < 0.05, and **(HT+MEL), ^^(HT+Epo) where *P* < 0.01.

aEEG data were available for all but one piglet (HT+Epo) and shown in Fig. 2. Hourly background cerebral electrical activity was categorized and scored according to Hellstrom-Westas *et al.* (1995) by two team members blinded to treatment group and averaged over 6 h intervals. We observed significant improvement in aEEG scores from 25 to 30 h in both *double therapy groups* compared to HT+V [HT+MEL (LS means score difference 1.0, 95% CI (0.2–1.8), *P* = 0.02] and HT+Epo [difference 0.9, 95% CI (0.1–1.6), *P* = 0.033)]. Combined HT+MEL+Epo *triple therapy* was associated with aEEG recovery at 55–60 h [difference 0.8, 95% CI (0.03–1.7), *P* = 0.042]. We observed no difference in aEEG score in HT+MEL+Epo *triple therapy* versus HT+MEL or HT+Epo *double therapies* (*P* > 0.05). Seven animals had electrographic seizures: three in HT+V (one treated with phenobarbitone 20 mg/kg at 21 h), two in HT+MEL (one treated with phenobarbitone 20 mg/kg at 21 h, and two in HT+MEL+Epo (one treated with phenobarbitone 20 mg/kg at 23 h) (*P* > 0.05). One animal in HT+V developed status epilepticus from 46 h after HI, which was not recognized and untreated.

### ^1^H magnetic resonance spectroscopy

**Figure 3:**
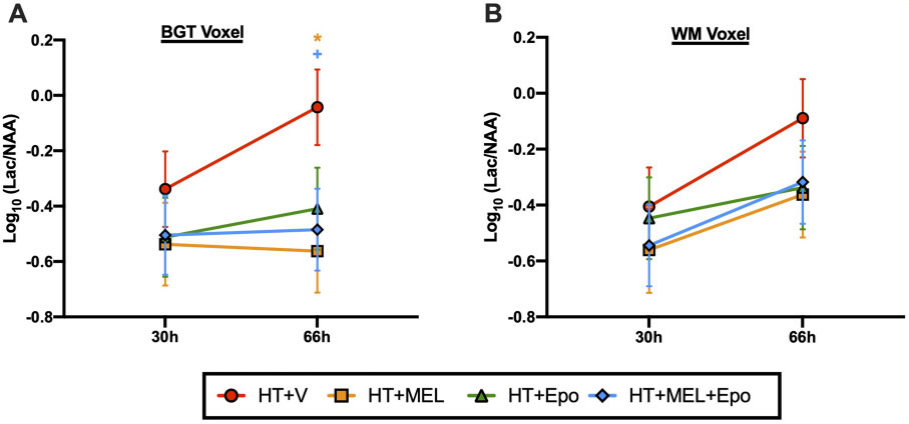
**^1^H MRS lactate/N-acetyl aspartate (Lac/NAA) peak ratios at 30 h and 66 h after HI.** Lac/NAA ratios were obtained for the BGT (**A**) and WM (**B**) voxels. An ANOVA model was fitted as previous described. Data presented are the grouped least square means Lac/NAA peak ratio ± standard error of the means (SEM) on the log_10_ scale. Comparison between treatment groups were assessed using 95% confidence intervals for Log_10_ (difference in least square means) and *P* values. Statistical significance shown as *(HT+MEL) and ^+^(HT+MEL+Epo) where *P* < 0.05 compared to HT+V.

^1^H MRS data were available for 42 piglets: HT+V *n* = 12, HT+MEL *n* = 10, HT+Epo *n* = 10, HT+MEL+Epo *n* = 10 and shown in Fig. 3. At 66 h, BGT Lac/NAA was reduced with HT+MEL [log_10_ LS means difference 0.52, 95% CI (0.12–0.92), *P* = 0.012] and HT+MEL+Epo [difference 0.442, 95% CI (0.04–0.84), *P* = 0.032] but not HT+Epo (*P* = 0.073) compared to HT+V. No difference in Lac/NAA was observed between treatment groups at 30 h in the BGT voxel, and at both 30 h and 66 h in the WM voxel. There was no difference between *double* and *triple therapy* groups in BGT and WM Lac/NAA at all-time points (*P* > 0.05).

### Immunohistochemistry

**Figure 4:**
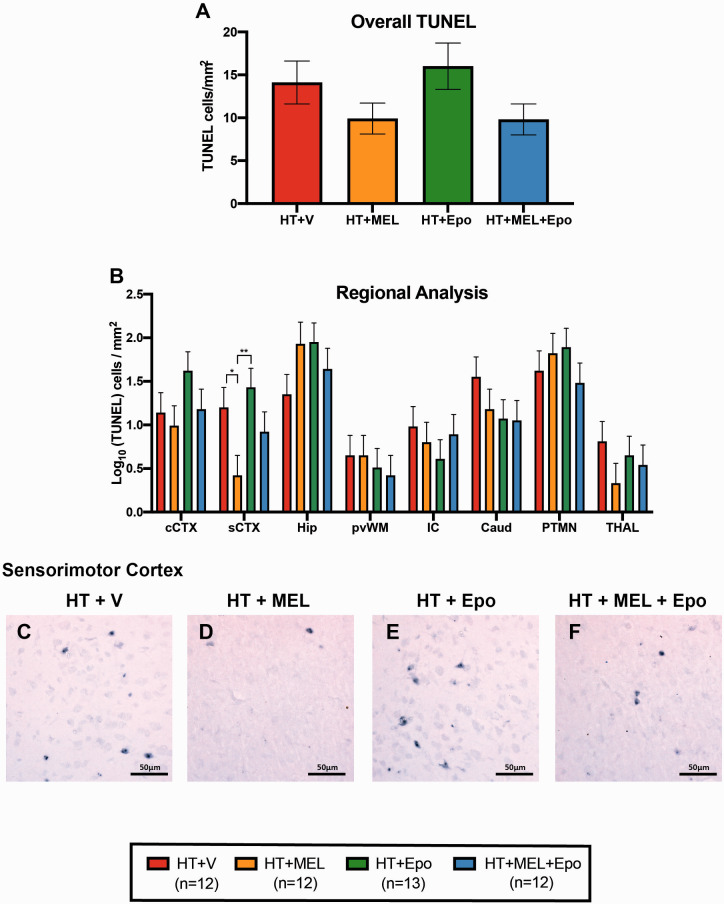
**TUNEL immunohistochemistry.** Overall (**A**) and regional (**B**) TUNEL-positive counts presented as least square means derived from an ANOVA model ± standard error of means (SEM) on the log_10_ scale. Example micrographs showing TUNEL-positive cells in sCTX with HT+V (**C**), HT+MEL (**D**), HT+Epo (**E**) and HT+MEL+Epo (**F**). Differences between groups were examined using 95% confidence intervals for the log_10_ (difference in least square means) and p values. Significant differences shown as *P < 0.05 and **P < 0.01. CAUD = caudate nucleus; cCTX = cingulate gyrus; HIP = hippocampus; IC = internal capsule, PTMN = putamen; PvWM = periventricular white matter; sCTX = sensorimotor cortex; THAL = thalamus.

**Figure 5:**
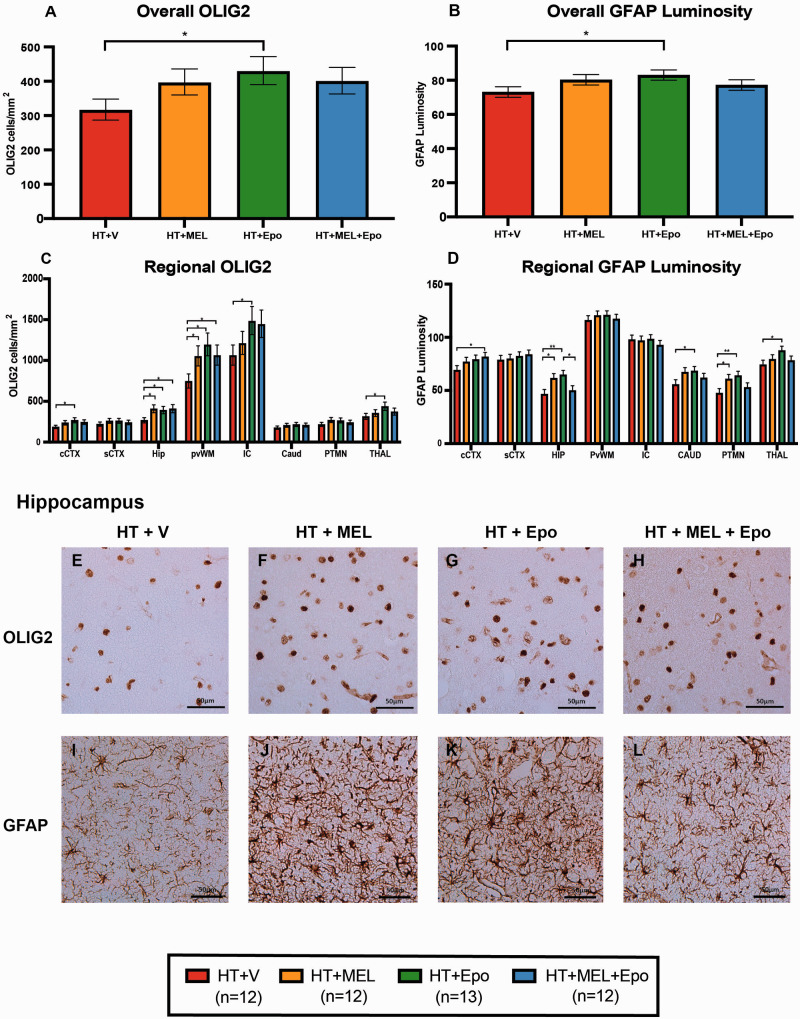
**Immunohistochemistry for oligodendrocytes (OLIG2) and astrocytes (GFAP luminosity).** OLIG2 cell counts and GFAP luminosity were fitted to the ANOVA model. The geometric means ± standard error of means (SEM) are shown for the overall (**A**) and regional (**C**) OLIG2 cell counts/mm^2^ after back transformation from the log_10_ scale. Mean overall (**B**) and regional (**D**) GFAP luminosity are also plotted with SEM error bars. Example micrographs from the HIP are shown in (**E–L**). Differences between groups were examined using 95% confidence intervals for difference in the least square means and significant differences between groups are shown *P < 0.05 and **P < 0.01. CAUD = caudate nucleus; cCTX= cingulate gyrus; HIP = hippocampus; IC = internal capsule; PTMN = putamen; PvWM = periventricular white matter; sCTX = sensorimotor cortex; THAL = thalamus.

Immunohistochemistry data from eight brain regions were available for 49 animals. The least square mean data of TUNEL-positive cells/mm^2^, OLIG2-positive cells/mm^2^ and GFAP luminosity are shown in Figs 4 and 5.

#### Terminal deoxynucleotidyl transferase-mediated deoxyuridine triphosphate nick-end labelling

Regarding overall TUNEL counts, compared to HT+V, we observed no difference in TUNEL-positive cells in HT+MEL [log_10_ LS means difference 0.148, 95% CI (−0.29 to 0.59), *P* = 0.498], HT+Epo [difference 0.053, 95% CI (−0.38 to 0.48), *P* = 0.805] and HT+MEL+Epo [difference 0.151, 95% CI (−0.29 to 0.59), *P* = 0.490] (Fig. 4A). In regional analysis, we observed a reduction in TUNEL-positive cell counts in the sensorimotor cortex (sCTX) with HT+MEL *double therapy* compared to HT+V [difference 0.788, 95% CI (0.14–1.43), *P* = 0.017]. TUNEL-positive cells were also reduced comparing HT+MEL to HT+Epo [difference 1.01, 95% CI (0.38–1.64), *P* = 0.002]. There was no difference between HT+MEL and HT+MEL+Epo (*P* = 0.392).

#### Oligodendrocyte transcription factor 2

HT+Epo *double therapy* was associated with an overall increase in surviving OLIG2-positive cells compared to HT+V [ratio of geometric LS means difference 1.36, 95% CI (1.03–1.78), *P* = 0.029] (Fig. 5). On regional analysis, we observed higher OLIG2 positive cells in five regions in HT+Epo versus HT+V: the cingulate cortex (ratio 1.44, 95% CI 1.04–2.0, *P* = 0.029), HIP (ratio 1.46, 95% CI 1.05–2.04, *P* = 0.024), periventricular white matter (PvWM) (ratio 1.60, 95% CI 1.15–2.22, *P* = 0.005), internal capsule (ratio 1.4, 95% CI 1.01–1.94, *P* = 0.046) and thalamus (ratio 1.39, 95% CI 1.0–1.93, *P* = 0.047). We also observed higher OLIG2-positive cells in 2 regions in HT+MEL versus HT+V: the HIP (ratio 1.52, 95% CI 1.09–2.13, *P* = 0.014) and PvWM (ratio 1.41, 95% CI 1.02–1.96, *P* = 0.039). HT+MEL+Epo versus HT+V was associated with increased OLIG2-positive cells in two regions: the HIP (ratio 1.53, 95% CI 1.09–2.14, *P* = 0.014) and PvWM (ratio 1.43, 95% CI 1.03–1.98, *P* = 0.034) but no difference was observed when compared with HT+Epo or HT+MEL in all regions of the brain.

#### GFAP luminosity

We observed an overall increase in GFAP luminosity in HT+Epo versus HT+V (LS means difference 9.9, 95% CI 1.2–18.6, *P* = 0.026) (Fig. 5). HT+Epo versus HT+V was associated with increased luminosity in four regions of the brain: HIP (difference 18.2, 95% CI 5.8–30.5, *P* = 0.004), caudate (difference 12.6, 95% CI 0.4–24.8, *P* = 0.043), putamen (PTMN) (difference 16.6, 95% CI 4.5–28.6, *P* = 0.007) and thalamus (difference 13.4, 95% CI 1.3–25.4, *P* = 0.03). We also observed higher luminosity in HT+MEL treated animals versus HT+V in 2 regions of the brain: the HIP (difference 15.0, 95% CI 2.4–27.7, *P* = 0.02) and PTMN (difference 13.4, 95% CI 1.1–25.7, *P* = 0.033). Treatment with HT+MEL+Epo was associated with increased GFAP in cingulate cortex compared to HT+V (difference 12.5, 95% CI 0.2–24.8, *P* = 0.046) and reduced GFAP luminosity compared with HT+Epo in the HIP (14.8, 95% CI 2.2–27.4, *P* = 0.022). No other significant differences were observed between HT+MEL+Epo versus HT+Epo or HT+MEL.

Results for the aEEG, MRS and immunohistochemistry outcome measures are summarized in Table 2.

**Table 2:** Summary of findings

	Amplitude-integrated EEG	Proton MRS Lac/NAA peak ratio	Immunohistochemistry		
			TUNEL-positive cells	OLIG2-positive cells	GFAP luminosity
HT+MEL *double therapy* versus HT+V	**Improved recovery from 25 to 30 h**	**Ameliorated BGT Lac/NAA rise at 66 h**	**↓ in sCTX**	**↑ 2 regions: HIP**; **PvWM**	**↑ 2 regions: HIP**; **PTMN**
HT+Epo *double therapy* versus HT+V	**Improved recovery from 25 to 30 h**	No significant difference	No significant difference	**↑ 5 regions: HIP, PvWM, cCTX, IC, THAL**	**↑ 4 regions: HIP, PTMN, CAUD, THAL**
HT+MEL+EPO *Triple therapy* versus HT+V	**Improved recovery from 55 to 60 h**	**Ameliorated BGT Lac/NAA rise at 66 h**	No significant difference	**↑ 2 regions: HIP, PvWM**	**↑ 1 region: cCTX**
*Triple therapy* versus *double therapies*	No significant improvement	No significant improvement	No significant difference	No significant difference	No significant difference

BGT = basal ganglia and thalamic voxel; CAUD = caudate nucleus; cCTX= cingulate gyrus; EEG = electroencephalogram; HIP = hippocampus; IC = internal capsule; Lac/NAA = lactate to *N*-acetyl aspartate; MRS = proton magnetic resonance spectroscopy; PTMN = putamen; PvWM = periventricular white matter; sCTX = sensorimotor cortex; THAL = thalamus.

Statistical significance (*P* < 0.05) highlighted in bold.

### Pharmacokinetics

**Figure 6:**
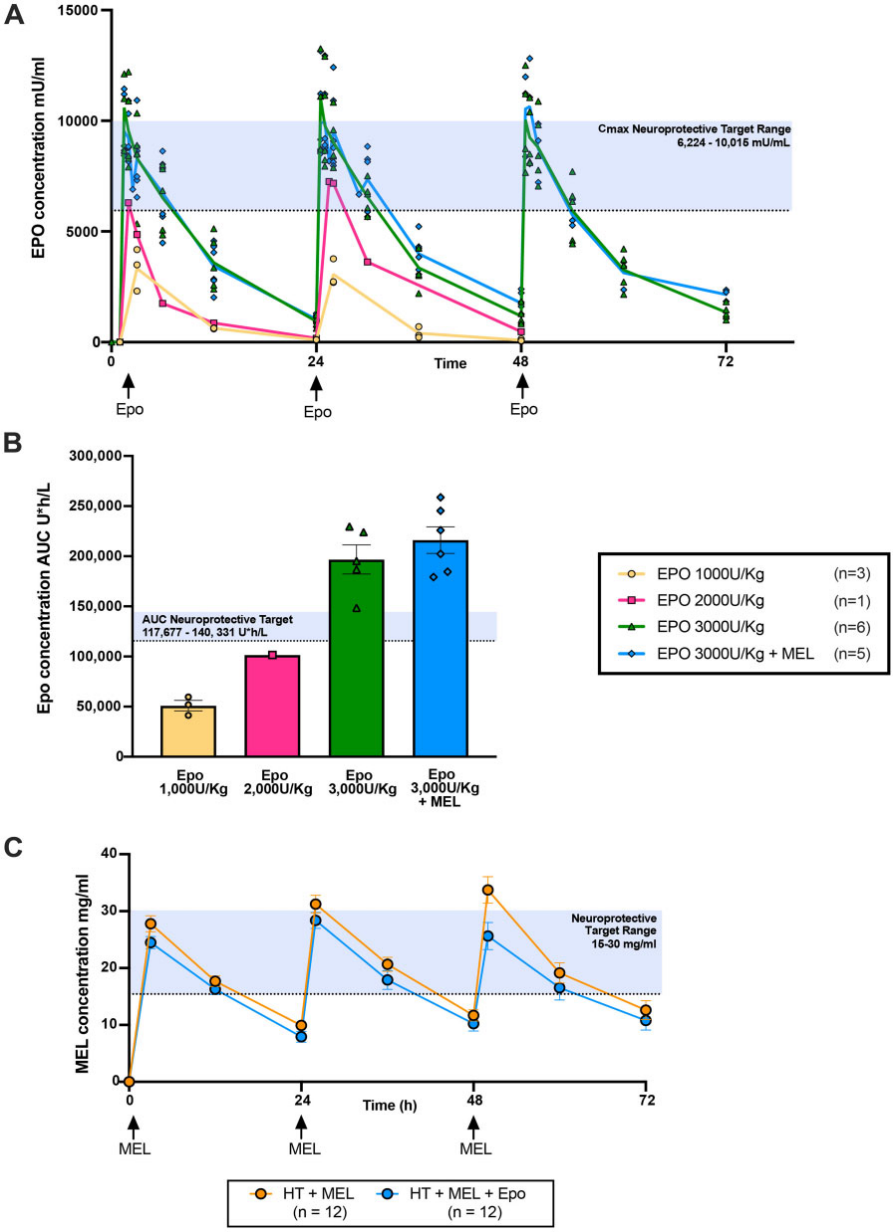
**Pharmacokinetic (PK) studies of Epo and melatonin in the piglet.** Previous pre-clinical studies identified the therapeutic target for Epo neuroprotection as: *C*_max_ 6224–10 015 mU/ml and AUC_48_ 117 677–140 000 U*h/l (Statler *et al.*, 2007). Epo at 1000 u/kg (*n* = 3), 2000 u/kg (*n* = 1) and 3000 u/kg [with (*n* = 5) and without (*n* = 6) MEL 20 mg/kg)] was administered as an intravenous bolus at 1 h, 24 h and 48 h and Epo levels as maximum concentration (*C*_max_) (Mean ± SEM) (**A**) and total AUC levels over 48 h (AUC_48_) (**B**) are illustrated. Mean (±standard deviation) melatonin (MEL) plasma concentration (mg/l) for HT+MEL (*n* = 12) and HT+MEL+Epo (*n* = 12) treated animals are shown in **C**. The therapeutic level for MEL is likely between 15 mg/l and 30 mg/l based on our previous studies (Robertson *et al.*, 2013, 2019, 2020).

Previous pre-clinical studies identified the therapeutic target for Epo neuroprotection as *C*_max_ 6224–10 015 mU/ml and AUC_48_ 117 677–140 000 U*h/l (Statler *et al.*, 2007). We performed pharmacokinetics (PK) studies to determine the dose to achieve this level. The PK profile of piglets given intravenous Epo boluses at 1000 U/kg (*n* = 3), 2000 U/kg (*n* = 1) and 3000 U/kg (with MEL *n* = 5, without MEL *n* = 6), 1 h, 24 h and 48 h after HI were assessed (Fig. 6). Epo at 3000 U/kg achieved a mean *C*_max_ (±SEM) of 9879 ± 436 mU/ml, 30 min after administration and mean AUC_48_ (±SEM) of 207 286 ± 32 521 U*h/l. The *C*_max_ was within the therapeutic range and was selected as the optimal dose for this study in piglets. There was no difference in *C*_max_ (*P* = 0.915) and AUC_48_ (*P* = 0.355) between animals treated with HT+Epo 3000 U/kg and HT+MEL+Epo 3000 U/kg.

Intravenous MEL infusion started 1 h after HI at 20 mg/kg given over 2 h resulted in plasma MEL levels achieving therapeutic range at 3 h after HI. In the HT+MEL group, the mean(±SEM) peak levels were: 27.8(±1.4) µg/ml, 31.2(±1.5) µg/ml and 33.7(±2.3) µg/ml at 2 h after the start of each MEL infusion (time points: 3 h, 26 h and 50 h after HI). AUC 0–72 h for each individual profile were mean 1407(±86) µg/ml*h and 1212(±350) µg/ml*h for HT+MEL. MEL levels between HT+MEL and HT+MEL+Epo were not significantly different (*P* = 0.182).

## Discussion

In this piglet model of neonatal HI, we observed some improvement in all pre-defined outcome measures with HT+MEL *double therapy* compared to HT+V. HT+MEL was associated with (i) more rapid aEEG recovery from 25 h to 30 h, (ii) amelioration of Lac/NAA rise in the BGT region at 66 h, (iii) reduction in TUNEL-positive cells in the sCTX; and (iv) improved oligodendrocyte cell survival in the HIP and PvWM and increased GFAP luminosity in HIP and PTMN. HT+Epo *double therapy* had a different pattern of protection with (i) more rapid aEEG recovery from 25 h to 30 h, (ii) increase in oligodendrocyte survival in five of eight brain regions and GFAP luminosity over five of eight brain regions. There was no effect of HT+Epo on MRS or TUNEL-positive cells, reflecting the different targets and effects of Epo and MEL therapy. HT+MEL+Epo (*triple therapy*) showed added brain protection compared to HT+V with improved aEEG recovery, reduction in ^1^H MRS Lac/NAA at 66 h and increased oligodendrocyte survival. However, we did not observe increased protection with *triple* compared to *double therapy*. The different effects of MEL and Epo on HT protection, with a more pronounced effect of MEL on TUNEL cell death and Epo on oligodendrocyte survival, suggest complementary effects that might become more visible with longer-term studies.

The administration of MEL, Epo and MEL+Epo in combination with 12 h HT was safe and led to no difference in physiological parameters and inotropic requirements compared to HT+V. Therapeutic Epo levels were achieved within 30 min. Following PK exploratory studies, we used an Epo dose of 3000 U/kg every 24 h and achieved the maximal Epo concentrations (*C*_max_) and exposure levels (AUC) similar to those associated with neuroprotection in other pre-clinical (Statler *et al.*, 2007) and clinical studies (Wu *et al.*, 2012, 2016). In infants, doses of 1000 U/kg every 48 h was sufficient to achieve therapeutic levels however doses up to 3000 U/kg are safe (Fauchere *et al.*, 2008). *In vitro* (Carloni *et al.*, 2018) and piglet studies (Robertson *et al.*, 2019) suggest the therapeutic level of MEL is ∼15–30 mg/l. Our experience from published studies (Robertson *et al.*, 2013, 2019, 2020) suggests that MEL neuroprotection is dependent on: (i) the time after HI to achieve therapeutic levels and (ii) the duration of exposure within a 24 h period to therapeutic MEL levels. In a previous study, we used a longer MEL infusion time of 6 h with 24 h HT; however, the delay of 8 h for MEL to reach therapeutic levels reduced the potential for protection (Robertson *et al.*, 2019). In this current study with 12 h cooling, therapeutic MEL levels were achieved by 3 h after HI and remained in a range for 12–18 h after each infusion in both MEL groups. Optimizing both HT and the duration of therapeutic levels of MEL are likely to increase protection. Indeed, the most widespread protection we observed, in both the white and deep grey matter was with 30 mg/kg MEL in ethanol vehicle given at 10 min after HI with 24 h HT (2–26 h after HI); MEL achieved therapeutic levels within 1 h of HI and these levels were maintained for most of the study (Robertson *et al.*, 2013). This may reflect more optimal dosing of MEL (Robertson *et al.*, 2013) combined with a longer cooling period compared to this current study. A more severe injury is also likely to expand the potential for brain protection with HT and MEL; based on the regional TUNEL-positive cell counts (mean cells/mm^2^), the injury was more severe in Robertson *et al.* (2020) than in the current study, particularly in the internal capsule. This may explain why, despite the larger group size in the current study, that brain protection with MEL was modest. Taken together (Robertson *et al.*, 2013, 2019, 2020), it is likely that MEL-augmented HT would be effective in severely affected brain regions, with the potential to protect both white and grey matter.

MEL and Epo have diverse mechanisms of action, targeting different parts of the cascade of injury after HI. Multiple signalling pathways are activated with HI, leading to oxidative stress, Ca^2+^ dys-homeostasis, mitochondrial dysfunction, activation of pro-inflammatory mediators, excitotoxicity and programmed cell death. Understanding this cascade of molecular events is important to develop new therapeutic strategies for NE. This need for early therapeutic levels of MEL aligns with its diverse anti-oxidative mechanisms preventing free radical-induced oxidative damage to the electron transport chain and mitochondrial DNA (Lopez *et al.*, 2009) as well as protection against excitotoxic damage (Husson *et al.*, 2002). MEL also exerts its effects through the downregulation of the pro-inflammatory transcription factor, NFĸB thereby reducing neuroinflammation. Mitochondrial integrity is preserved through the stabilization and protection from nitro-oxidative damage to membrane lipids and inhibiting pro-apoptotic proteins (Andrabi *et al.*, 2004). MEL stimulates neuronal differentiation and cell proliferation, which are critical in neuroplasticity. Like MEL, Epo has anti-apoptotic, anti-inflammatory, neurotrophic and antioxidant properties; however, Epo also promotes brain repair through angiogenesis, oligodendrogenesis and neurogenesis (Juul and Pet, 2015).

Amplitude-integrated EEG and ^1^H MRS are validated (Azzopardi *et al.*, 2019; Lally *et al.*, 2019; Mitra *et al.*, 2019) translational biomarkers used in the clinical setting for infants with NE. Here, we showed *double therapy* with HT+MEL and HT+Epo was associated with more rapid aEEG recovery than H + V. In the clinical setting, aEEG recovery after HI predicts outcomes in infants with NE (Thoresen *et al.*, 2010; Csekő *et al.*, 2013; Chandrasekaran *et al.*, 2017). Surprisingly, *triple therapy* (HT+MEL+Epo) led to a slower recovery of aEEG (∼24 h later) than *double therapy.* It is unclear why Epo might negatively interact with MEL to delay aEEG recovery by 24 h, especially as both *double therapy* arms (including that of HT+Epo) showed similar aEEG recovery trajectories. Further work is needed to understand specific interactions between MEL and Epo in the early part of the evolving cascade of injury after HI. These aEEG data may support future study protocols to stagger the administration of adjunct therapies, with early MEL and later Epo (after HT). Interestingly, both treatment arms with added MEL (HT+MEL and HT+MEL+Epo) were associated with an amelioration of the rise in BGT Lac/NAA at 66 h. BGT Lac/NAA is the most accurate magnetic resonance imaging biomarker currently to predict neurodevelopmental outcomes (Thayyil *et al.*, 2010; Mitra *et al.*, 2019) and has been successfully used (Azzopardi *et al.*, 2016) and subsequently validated (Azzopardi *et al.*, 2019) as a surrogate outcome measure in a clinical neuroprotection study in babies.

The localized reduction in TUNEL-positive cell counts observed in the sCTX in HT+MEL treated animals concurs with our previous study (Robertson *et al.*, 2019) using the same proprietary MEL formulation with 15 mg/kg given over 6 h and started at 2 h. Although study durations differed (48 h versus 72 h in this study), the same region was specifically protected with no significant protection in other brain regions. In the piglet brain, the most vulnerable regions to HI are the motor and somatosensory cortex together with the sensorimotor-recipient region of the central PTMN (Martin *et al.*, 1997). These regions are components of the newborn telencephalon, which are areas of increased metabolic demand including high glucose consumption and mitochondrial activity (Martin *et al.*, 1997). Such localized protection of the sCTX would lead to an important improved motor function in babies which might be detectable at 18 months to 2 years in survivors of NE. Protection in the sCTX was seen only with the proprietary MEL formulation [current study and (Robertson *et al.*, 2019)] and not observed in the studies using ethanol excipient where protection was seen in areas which sustained the most damage (mainly WM and internal capsule in Robertson *et al.*, 2020 and both white and deep grey matter in Robertson *et al.*, 2013). The variable effect of ethanol excipient, with partial protection on the one hand (Robertson *et al.*, 2020) and suppression of cell proliferation and microglial activation in a foetal sheep model on the other (Drury *et al.*, 2014) complicates the picture. The regional protection in the sCTX with proprietary MEL formulation needs further close study to assess the clinical potential and relevance.

Treatment with HT+Epo *double therapy* was associated with increased oligodendrocyte survival, even though we did not observe reduced TUNEL-positive cells. Protection was highest in the PvWM and internal capsule, where cell counts were approximately 1.5 times higher in HT+Epo *double therapy* compared to HT+V. Clinically this would translate into improved motor (Martinez-Biarge *et al.*, 2011) and cognitive outcomes. Oligodendrocytes are highly susceptible to HI injury from glutamate toxicity, free radical-mediated damage and inflammation (Kaur *et al.*, 2013). Epo acts through several pathways to improve cell survival. Epo inhibits the transcription of iNOS mRNA responsible for NO production and oxidative stress (Genc *et al.*, 2006). Via the Epo receptor-dependent pathway, Epo activates Jak2 kinase leading to NFkB and Stat5 mobilization into the nucleus and gene expression of the anti-apoptotic factors, bcl and bcl-xl (Juul and Pet, 2015). Epo also reduces the expression of Fas/FasL which are part of the extrinsic apoptotic pathway (Huang *et al.*, 2019). Epo upregulates genes responsible for myelination in oligodendrocytes (Gyetvai *et al.*, 2017) and promotion of oligodendrogenesis, which enhances oligodendrocyte survival and enhances myelin production (Jantzie *et al.*, 2013). Interestingly, despite no preservation of brain volume following HI in Epo treated Day-7 rats, improvement in sensorimotor function was observed 14 days after injury (Iwai *et al.*, 2010), coinciding with increased proliferation and maturation of oligodendrocyte precursor cells and restoration of myelinated axons in the cortex and striatum.

Increased GFAP luminosity, a marker of reactive astrogliosis was seen in the HIP and PTMN with *double therapies* (HT+MEL and HT+Epo). GFAP luminosity was also increased in the caudate and thalamus of HT+Epo groups. In our previous studies, we saw no difference in GFAP luminosity with lower doses (5 or 15 mg/kg) proprietary MEL (Robertson *et al.*, 2019) but some increases in GFAP luminosity with 18 mg/kg MEL, although some of these changes may have been driven by the ethanol excipient (Robertson *et al.*, 2020). In other animal studies, reduction in astrogliosis with MEL (Babaee *et al.*, 2015) or Epo (Hwang *et al.*, 2019) have been reported. The reasons for these differences are unclear. *In vitro* studies (Sugawa *et al.*, 2002; Gonzalez *et al.*, 2007) show Epo induces astrocyte proliferation in a dose-dependent manner. MEL (Radogna *et al.*, 2009) and Epo (Liu *et al.*, 2006) also protect astrocytes from oxidative stress injury, therefore our findings of increased GFAP luminosity may be partially explained by improved survival in the astrocyte population. This is supported by our findings in the PvWM and internal capsule which had the highest GFAP luminosity but also the highest proportion of oligodendrocytes and lowest TUNEL-positive cell counts. Another explanation may be that, at 72 h, brain injury and protection are still evolving. Although astrogliosis is associated with pro-inflammatory cytokine release, exacerbating brain injury, astrocytes also aid neuronal recovery (Bylicky *et al.*, 2018) and oligodendrocyte survival (Kato *et al.*, 2011) following injury. Astrocytes release anti-oxidant enzymes and buffer extra-cellular glutamate to reduce excitatory neuronal damage (Liu and McCullough, 2013). Thus, the balance between the detrimental and neuroprotective effects of astrogliosis remains unclear.

There are limitations to our study. To reduce variability, we used male piglets, preventing investigation of the impact of sex on neuroprotection. Although we extended our study duration to 72 h from 48 h (Robertson *et al.*, 2013, 2019, 2020), the possible long-term neuroregenerative benefit of adding Epo to MEL may become more apparent beyond 72 h. Epo stimulates neurogenesis (Shingo *et al.*, 2001; Gonzalez *et al.*, 2007; Osredkar *et al.*, 2010) and the migration of neuronal progenitors to the site of injury (Wang *et al.*, 2006; Iwai *et al.*, 2007); such regenerative properties may not be detectable on MRS and histology at 72 h, only becoming apparent weeks after HI. Unfortunately extending the study beyond 72 h is not feasible due to confounding effects with longer anaesthetic exposure (Broad *et al.*, 2016) and evolving multiorgan failure. The outcomes from two large multicentre randomized clinical trials (University of Sydney National Health and Medical Research Council Australia, 2016; Juul *et al.*, 2018) investigating the efficacy of Epo to augment HT are awaited. In this study, animals underwent 12 h HT to model the sequence of cooling, rewarming and MRS acquisition at two time points during normothermia, similar to the clinical setting. Protection after HI is related to the duration of cooling in pre-clinical (Davidson *et al.*, 2015, 2016) and clinical studies (Shankaran *et al.*, 2017), and thus we cannot exclude the possibility of a different effect of 12 h versus 72 h cooling in babies with NE. Longer duration studies are therefore needed; we are developing a 5-day survival model in which longer HT protocols are feasible with motor and behavioural assessment on Day 5. We observed that one subject in the HT+V group was in status epilepticus; this was detected retrospectively and is likely to have led to a secondary exacerbation of brain injury, as prolonged seizures are associated with increased histological injury (Bjorkman *et al.*, 2010). Our model used targeted cerebral ischaemia in association with global hypoxia, thus limiting systemic injury and acidosis. The post-HI blood pH was therefore higher than the pH < 7.0 threshold for HT in newborns. The base excess in the HT+Epo group just after HI was slightly higher than HT+MEL and HT+MEL+Epo groups; however, other post-HI parameters such as lactate and pH were similar. Four animals died in the first 24 h so outcome data were not available. Due to the exploratory nature of the study, no adjustments were made for multiple testing.

This study in the newborn piglet shows MEL and Epo are safe when administered with HT and are complementary therapies. HT+MEL *double therapy* showed modest but consistent beneficial effects across aEEG, Lac/NAA and immunohistochemistry; in future studies an increase in the MEL dose to 30 mg/kg/24 h may increase exposure to therapeutic levels and further improve protection. HT+Epo *double therapy* had the most effect on oligodendrocyte survival; this concurs with Epo’s effect on regeneration and indicates longer-term studies are needed to assess its full potential. HT+MEL+Epo *triple therapy* did not augment brain protection further from that seen with *double therapies.* Staggering the administration of therapies with early MEL and later Epo (after HT) may provide better protection; each therapy has complementary actions which may be time critical during the neurotoxic cascade after HI. Clinical trial results of HT+Epo are awaited and clinical trials to assess HT+MEL are needed.

## Supplementary material

Supplementary material is available at *Brain Communications* online.

## Supplementary Material

fcaa211_Supplementary_Data

## Abbreviations

^1^H =: proton
aEEG =: amplitude-integrated electroencephalogram
AUC =: area under the curve
BD =: bis in die (twice a day)
BGT =: basal ganglia, thalamus
CAUD =: caudate nucleus
cCTX =: cingulate gyrus
Epo =: erythropoietin
GFAP =: glial fibrillary acidic protein
HI =: hypoxia–ischaemia
HIP =: hippocampus
HT =: hypothermia
IC =: internal capsule
Lac/NAA =: lactate/*N*-acetyl aspartate
MABP =: mean arterial blood pressure
MEL =: melatonin
MRS =: magnetic resonance spectroscopy
NE =: neonatal encephalopathy
OD =: omne in die (once a day)
OLIG2 =: oligodendrocyte transcription factor 2
PK =: pharmacokinetics
PTMN =: putamen
sCTX =: sensorimotor cortex
PvWM =: periventricular white matter
THAL =: thalamus
TUNEL =: terminal deoxynucleotidyl transferase-mediated deoxyuridine triphosphate nick-end labelling
V =: vehicle
WM =: white matter
