# Supplementary material for: Melatonin and/or erythropoietin combined with hypothermia in a piglet model of perinatal asphyxia
Source: Brain Commun. 2020 Dec 1;3(1):fcaa211. doi: 10.1093/braincomms/fcaa211 (PMC7876304; doi:10.1093/braincomms/fcaa211)
Supplement: fcaa211_Supplementary_Data [file fcaa211_supplementary_data.docx]

**Supplementary Materials and Methods**

**Supplementary Fig 1: Histology of the piglet brain.** Hemotoxylin and Eosin stained section of the piglet brain showing the 8 regions assessed at 2 levels using 3 fields per region. 1. Cingulate cortex, 2. Sensorimotor cortex, 3. Hippocampus (1 level only), 4. Periventricular white matter, 5. Internal capsule, 6. Caudate nucleus, 7. Putamen, 8. Thalamus

**Supplementary Table 1: Grouped physiological data through the duration of the experiments.**  With the exception of inotropes, data are presented as least square means (LS means) with 95% confidence intervals (CI). LS means deduced from an ANOVA model fitted with fixed factor effects of treatment, time interval and treatment*time interval interaction, plus a random effect subject to take account of repeated measures. Comparison between treatment groups were assessed with one-way ANOVA across the 4 treatment groups where * indicates p<0.05. Post-hoc comparison between groups using 95% confidence intervals for difference in the least square means. ^Inotropes data are presented as median dose averaged over the 72h experiments, with interquartile ranges and comparisons were made between groups using Kruskal-Wallis test.

|  |  | **HT + V** | | | **HT + MEL** | | | **HT + Epo** | | | **HT + MEL + Epo** | | | p value |
| --- | --- | --- | --- | --- | --- | --- | --- | --- | --- | --- | --- | --- | --- | --- |
|  |  | LS Mean | Lower 95% CI | Upper 95% CI | LS Mean | Lower 95% CI | Upper 95% CI | LS Mean | Lower 95% CI | Upper 95% CI | LS Mean | Lower 95% CI | Upper 95% CI |  |
| Weight (kg) | | **2.0** | 1.9 | 2.1 | **1.9** | 1.8 | 2.0 | **2.0** | 1.9 | 2.1 | **2.0** | 1.9 | 2.1 | 0.517 |
| Rectal Temperature (°C) | Baseline | **38.0** | 37.8 | 38.1 | **37.9** | 37.8 | 38.1 | **37.8** | 37.6 | 37.9 | **38.0** | 37.9 | 38.2 | 0.616 |
|  | 0-1h | **38.1** | 38.0 | 38.3 | **38.0** | 37.9 | 38.2 | **38.0** | 37.9 | 38.2 | **38.1** | 37.9 | 38.2 | 0.216 |
|  | 1-25h | **34.9** | 34.8 | 35.1 | **35.0** | 34.8 | 35.1 | **35.0** | 34.9 | 35.1 | **34.9** | 34.8 | 35.0 | 0.060 |
|  | 25-49h | **38.1** | 38.0 | 38.2 | **38.0** | 37.9 | 38.2 | **38.0** | 37.8 | 38.1 | **38.0** | 37.9 | 38.1 | 0.100 |
|  | 49-72h | **38.0** | 37.9 | 38.1 | **38.0** | 37.9 | 38.2 | **38.0** | 37.9 | 38.1 | **38.0** | 37.9 | 38.1 | 0.984 |
| Inotropes^ | Dopamine (mcg/kg/min) | **9.1** | 0.8 | 10.6 | **7.6** | 6.9 | 13.3 | **6.2** | 3.0 | 9.0 | **6.9** | 4.4 | 16.9 | 0.355 |
|  | Dobutamine  (mcg/kg/min) | **0** | 0 | 3.1 | **0** | 0 | 5.1 | **0** | 0 | 0 | **0** | 0 | 3.6 | 0.525 |
|  | Adrenaline  (ng/kg/min) | **0** | 0 | 0 | **0** | 0 | 0 | **0** | 0 | 0 | **0** | 0 | 0.1 | 0.202 |
|  | Noradrenaline  (ng/kg/min) | **0** | 0 | 1.8 | **0** | 0 | 1.1 | **0** | 0 | 0 | **0** | 0 | 3.9 | 0.547 |
| Heart Rate (bpm) | Baseline | **166.0** | 154.3 | 177.7 | **159.4** | 148.0 | 170.7 | **164.4** | 153.5 | 175.3 | **173.3** | 161.9 | 184.6 | 0.273 |
|  | 0-1h | **198.7** | 187.4 | 210.0 | **191.5** | 180.1 | 202.8 | **188.5** | 177.7 | 199.4 | **189.5** | 178.2 | 200.8 | 0.518 |
|  | 1-25h | **178.2** | 166.8 | 189.5 | **169.0** | 157.7 | 180.4 | **165.1** | 154.2 | 176.0 | **174.7** | 163.3 | 186.0 | 0.476 |
|  | 25-49h | **181.8** | 170.5 | 193.2 | **168.6** | 157.3 | 180.0 | **175.0** | 164.2 | 185.9 | **176.3** | 164.9 | 187.6 | 0.531 |
|  | 49-72h | **157.9** | 146.6 | 169.3 | **151.6** | 140.3 | 162.9 | **155.4** | 144.5 | 166.3 | **159.3** | 148.0 | 170.7 | 0.763 |
| Mean Arterial BP (mmHg) | Baseline | **51.3** | 48.4 | 54.2 | **47.8** | 45.0 | 50.6 | **49.0** | 46.3 | 51.7 | **48.3** | 45.5 | 51.1 | 0.401 |
|  | 0-1h | **49.3** | 46.5 | 52.1 | **46.3** | 43.5 | 49.1 | **44.5** | 41.9 | 47.2 | **45.0** | 42.3 | 47.8 | 0.291 |
|  | 1-25h | **44.5** | 41.7 | 47.3 | **44.9** | 42.2 | 47.7 | **46.8** | 44.1 | 49.4 | **45.3** | 42.5 | 48.1 | 0.525 |
|  | 25-49h | **52.2** | 49.4 | 55.0 | **49.6** | 46.8 | 52.3 | **49.2** | 46.5 | 51.9 | **48.9** | 46.1 | 51.6 | 0.210 |
|  | 49-72h | **50.6** | 47.8 | 53.4 | **49.9** | 47.1 | 52.7 | **51.3** | 48.6 | 53.9 | **50.4** | 47.6 | 53.1 | 0.895 |
| Blood gas |  |  |  |  |  |  |  |  |  |  |  |  |  |  |
| pH | Baseline | **7.44** | 7.40 | 7.49 | **7.51** | 7.46 | 7.55 | **7.48** | 7.44 | 7.53 | **7.44** | 7.40 | 7.48 | 0.309 |
|  | 12h | **7.48** | 7.44 | 7.53 | **7.47** | 7.42 | 7.51 | **7.47** | 7.43 | 7.52 | **7.43** | 7.39 | 7.48 | 0.531 |
|  | 24h | **7.42** | 7.38 | 7.47 | **7.46** | 7.42 | 7.50 | **7.40** | 7.36 | 7.45 | **7.46** | 7.41 | 7.50 | ***0.033**** |
|  | 48h | **7.45** | 7.41 | 7.50 | **7.44** | 7.40 | 7.49 | **7.45** | 7.40 | 7.49 | **7.43** | 7.39 | 7.48 | 0.861 |
|  | 72h | **7.48** | 7.44 | 7.52 | **7.46** | 7.42 | 7.51 | **7.45** | 7.41 | 7.49 | **7.45** | 7.41 | 7.50 | 0.738 |
| pCO2 | Baseline | **6.2** | 5.6 | 6.8 | **5.5** | 4.9 | 6.2 | **5.6** | 5.0 | 6.2 | **6.1** | 5.5 | 6.8 | 0.596 |
| (kPa) | 12h | **6.0** | 5.3 | 6.6 | **6.2** | 5.6 | 6.8 | **5.5** | 4.9 | 6.1 | **6.5** | 5.9 | 7.1 | 0.301 |
|  | 24h | **6.0** | 5.4 | 6.7 | **6.2** | 5.6 | 6.9 | **6.4** | 5.8 | 7.0 | **6.1** | 5.4 | 6.7 | 0.615 |
|  | 48h | **5.5** | 4.9 | 6.2 | **6.1** | 5.5 | 6.8 | **5.4** | 4.8 | 6.0 | **6.0** | 5.3 | 6.6 | 0.132 |
|  | 72h | **5.3** | 4.7 | 5.9 | **5.9** | 5.2 | 6.5 | **5.4** | 4.8 | 6.0 | **5.4** | 4.8 | 6.1 | 0.500 |
| Base Excess | Baseline | **7.2** | 5.0 | 9.4 | **8.8** | 6.6 | 11.0 | **6.2** | 4.1 | 8.3 | **6.8** | 4.6 | 9.0 | 0.483 |
| (mEq/L) | 12h | **9.2** | 7.0 | 11.4 | **9.8** | 7.6 | 12.0 | **6.8** | 4.7 | 8.9 | **8.5** | 6.3 | 10.7 | 0.287 |
|  | 24h | **5.3** | 3.1 | 7.4 | **9.8** | 7.6 | 11.9 | **5.4** | 3.3 | 7.5 | **8.0** | 5.8 | 10.2 | ***0.016**** |
|  | 48h | **5.8** | 3.6 | 8.0 | **7.3** | 5.1 | 9.5 | **3.5** | 1.4 | 5.6 | **5.6** | 3.4 | 7.8 | 0.054 |
|  | 72h | **6.7** | 4.5 | 8.9 | **7.5** | 5.3 | 9.7 | **3.9** | 1.8 | 6.0 | **4.8** | 2.6 | 6.9 | 0.146 |
| Lactate | Baseline | **2.9** | 2.1 | 3.7 | **2.7** | 1.9 | 3.5 | **2.8** | 2.0 | 3.5 | **4.1** | 3.3 | 4.9 | 0.087 |
| (mmol/L) | 12h | **3.0** | 2.2 | 3.8 | **2.4** | 1.6 | 3.2 | **2.7** | 1.9 | 3.5 | **3.5** | 2.7 | 4.2 | 0.453 |
|  | 24h | **3.6** | 2.9 | 4.4 | **1.7** | 1.0 | 2.5 | **2.8** | 2.1 | 3.6 | **2.7** | 1.9 | 3.5 | 0.113 |
|  | 48h | **1.4** | 0.6 | 2.2 | **1.5** | 0.7 | 2.3 | **1.6** | 0.9 | 2.4 | **1.7** | 0.9 | 2.5 | 0.687 |
|  | 72h | **1.3** | 0.5 | 2.1 | **1.2** | 0.4 | 2.0 | **1.1** | 0.3 | 1.8 | **1.4** | 0.6 | 2.2 | 0.164 |
| Glucose | Baseline | **6.0** | 4.1 | 7.8 | **5.2** | 3.4 | 7.1 | **5.3** | 3.5 | 7.2 | **6.1** | 4.3 | 8.0 | 0.161 |
| (mmol/L) | 12h | **11.6** | 9.7 | 13.5 | **10.3** | 8.4 | 12.2 | **10.4** | 8.6 | 12.2 | **13.7** | 11.8 | 15.6 | 0.233 |
|  | 24h | **12.2** | 10.3 | 14.1 | **6.9** | 5.0 | 8.8 | **8.0** | 6.2 | 9.8 | **8.7** | 6.8 | 10.5 | 0.070 |
|  | 48h | **5.6** | 3.7 | 7.5 | **5.8** | 4.0 | 7.7 | **7.3** | 5.5 | 9.1 | **7.1** | 5.2 | 9.0 | 0.249 |
|  | 72h | **6.1** | 4.2 | 8.0 | **5.1** | 3.2 | 6.9 | **5.6** | 3.8 | 7.4 | **6.0** | 4.1 | 7.8 | 0.406 |

***Hematology Results:***

Hematology results are shown in Supplementary Fig 2. Haemoglobin, haematocrit, mean cell volume (MCV), platelets, packed cell volume and white cell count values decreased over the duration of the experiment compared with baseline levels. We observed a higher red cell count at 24h in HT+Epo group compared with the other groups (p<0.05) however this was not observed at other time points. At 48h, haematocrit and packed cell volume were higher in HT+MEL and HT+Epo compared to HT+V however this was not observed at other time points. We did not observe different hematological values at 72h in piglets who received HT+Epo compared to HT+V and HT+MEL (p>0.05). Regular blood sampling likely contributed to the gradual decline in haemoglobin, haematocrit and packed cell volume over the duration of the experiments.

**Supplementary Fig 2: Grouped haematological values for the duration of the experiments.** Values presented as least square means with standard error of means. Comparisons made with ANOVA and significant differences between groups highlighted as *p<0.05
